# Outcomes of AO/OTA C-type fractures of the distal humerus after open reduction and internal fixation with locking plate constructs in patients at least 65 years old

**DOI:** 10.1186/s12891-022-05431-5

**Published:** 2022-06-01

**Authors:** Kaarlo V. Kervinen, Mikko T. Salmela, Tuomas A. Lähdeoja

**Affiliations:** 1 Medical Faculty, University of Helsinki, Helsinki, Finland; 2 Finnish Centre for Evidence-Based Orthopaedics (FICEBO), Department of Orthopaedics and Traumatology, University of Helsinki and Helsinki University Hospital, Topeliuksenkatu 5, 00029 Helsinki, HUS Finland

**Keywords:** Distal humerus fracture, Locking plate, Osteosynthesis, Surgical treatment, Elderly, Oxford Elbow Score

## Abstract

**Background:**

Modern treatment options of distal humerus fractures of active elderly patients are osteosynthesis and total elbow arthroplasty. The evidence of outcomes of ORIF after AO/OTA C-type fractures mostly predates the adoption of locking plates. We evaluated the results of open reduction and internal fixation of these fractures treated exclusively with anatomic locking plates.

**Methods:**

A retrospective cohort of 39 patients aged 65 years or above with ORIF for AO/OTA C-type distal humerus fracture using locking plates was analysed. 23 provided follow-up data and 14 attended a follow-up visit. Primary outcome was the Oxford Elbow Score. Secondary outcomes were Mayo Elbow Performance Score, quickDASH, satisfaction, range of motion, complications and revision surgeries.

**Results:**

Mean Oxford Elbow Score pain was 83 (SD 17), Oxford Elbow Score function 83 (17) and Oxford Elbow Score social-psychological 79 (20). Mean total Oxford Elbow Score was 81 (15). Among the 14 patients who attended a follow-up visit, Mayo Elbow Performance Score was 85 (17), qDASH 19 (16), active arc of motion 119 (19) degrees. Mayo Elbow Performance Score and arc of motion were worse than on the healthy side. One patient had a serious deep infection. Eleven patients had at least one revision surgery, of which 6 were implant removals and 2 subsequent total elbow arthroplasties.

**Conclusions:**

Distal AO/OTA C-type distal humerus fractures in older adults can be treated reliably and with good outcomes with ORIF using modern locking plates. The mean qDASH scores are similar to population normal values, but when compared to the healthy arm, single-arm outcomes indicated somewhat impaired function. About 1 in 4 patients had at least one revision surgery.

## Background

Fractures of the distal humerus in old adults are challenging due to poor bone quality and frequent fracture comminution. Modern treatment options include open reduction and internal fixation (ORIF) with locking plates and total elbow arthroplasty (TEA) which can restore joint function and stability [1, 2]. The surgical outcomes have been reported to be similar in ORIF and TEA, but the complication profiles differ [3]. Revision surgeries are commonly reported following ORIF, while deep infections after TEA are difficult to manage and many surgeons recommend a permanent limb loading limitation after a TEA [2, 3]. Nonsurgical treatment with cast immobilisation, also known as a “Bag of bones” treatment, has been used in elderly low-demand patients with acceptable outcomes considering the patient group [4].

The current literature on outcomes after ORIF in older adults are largely patient series where non-locking plates have been used [3]. Biomechanical studies have shown that in poor quality bone, locking plates provide a more rigid fixation than non-locking constructs. The options available with modern anatomic plates technically allow ORIF of almost all types of distal humerus fractures [5]. The outcomes in general adult population support ORIF as the first line treatment, but in the older population there is a paucity of evidence of outcomes with modern implants to guide treatment choices [6]. The age limit of “elderly” or “older adult” varies in studies. In Finland most people have retired from active work life by 65 years, making 65 years a natural choice for “elderly” or “older adult” in the Finnish population [3].

The purpose of the study was to evaluate the results of ORIF of AO/OTA C-type (complete articular) distal humerus fractures treated exclusively with anatomic locking plates, in patients at least 65 years of age at the time of injury.

## Methods

This was a retrospective cohort study with a patient file review and a clinical or telephone follow-up, after a minimum of 1 year after the injury. The study was approved by the Helsinki and Uusimaa Hospital District ethics committee (HUS/938/2017). Patients attending follow-up gave a written informed consent and all the methods were carried out in accordance with the Helsinki declaration. We used the STROBE statement as a guide [7].

### Eligibility criteria

Inclusion criteria: At least 65 years at old on the day of the injury; AO/OTA C-type distal humerus fracture; ORIF in Helsinki University Hospital (a large academic trauma tertiary centre serving a population of 1.5 M) between June 2009 and May 2016; minimum follow-up of one year after surgery and the ability to answer questionnaires in Finnish. We excluded patients with pathologic fractures and patients with inflammatory joint disease affecting the elbow. Anatomic locking plates had been adopted to routine use by the start of study period. TEA patients were not included. To be treated with osteosynthesis, the fractures had to appear “reducible” by the responsible surgeon. At the time, very distal and comminuted fractures were at the surgeon’s discretion sometimes treated with primary TEA. We did not include TEAs in this study as the number of TEAs were very small. Also, the fractures treated with TEA were too comminuted to be amenable to ORIF – hence the patient groups are fundamentally not comparable due to different fracture characteristics.

### Patient accrual

**Fig. 1:**
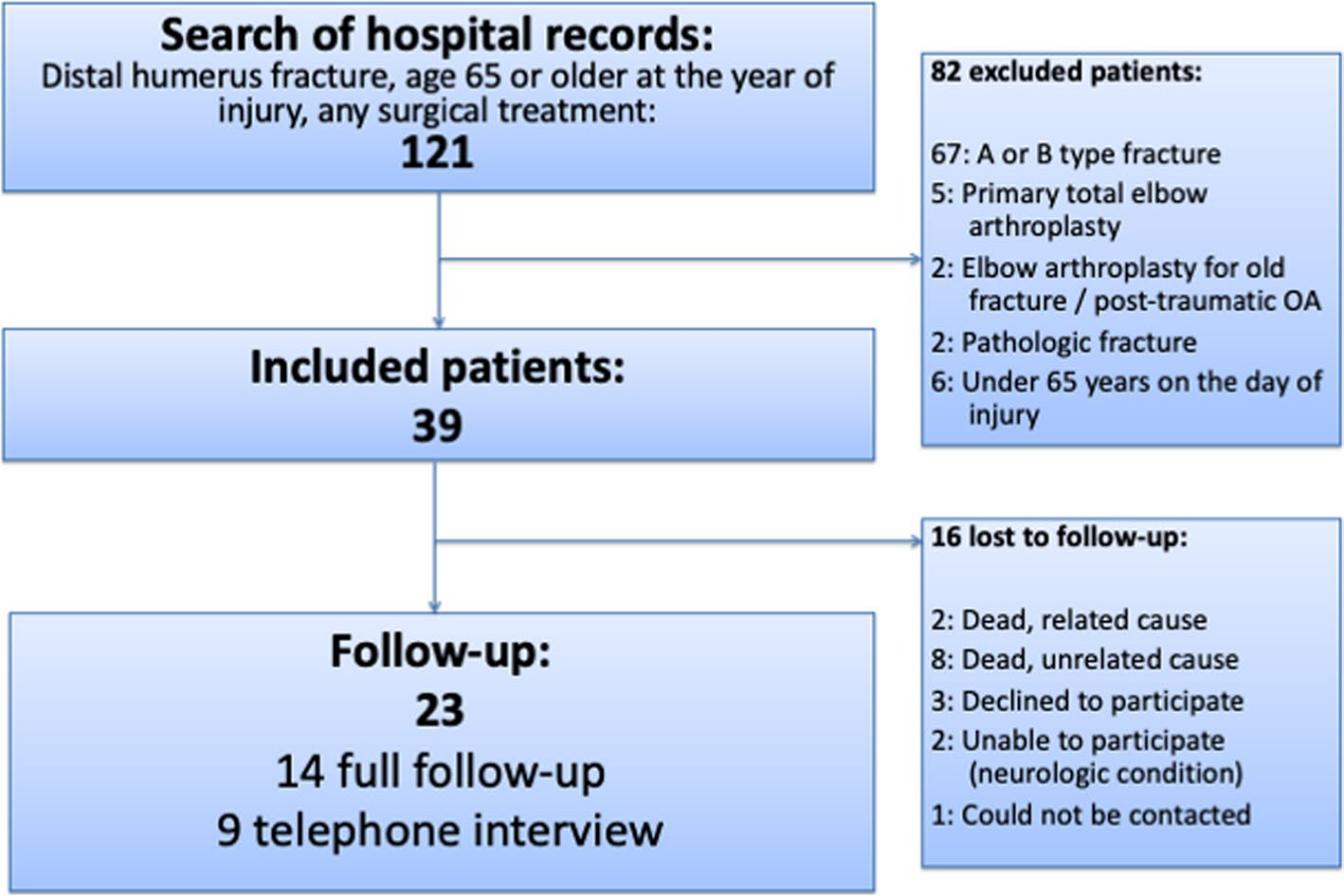
The study flowchart

Potentially eligible patients were identified from the electronic operating room database, using appropriate ICD-10 code (S42.4) with any surgical procedure code. The hospital records and patient radiographs were reviewed in April 2017 by KK and MS to identify eligible patients (Fig. 1). The patient and injury details, course and method of treatment, interventions and adverse events, clinical progression on outpatient visits after the injury, possible date and cause of injury-related death were extracted from electronic medical records.

Contact details of the patients were obtained from hospital records, national population registry and telephone directories. The patients were approached by letters and telephone. The patients were asked to attend a follow-up appointment for outcome measurements and a radiograph. Those unwilling or unable to visit were asked to participate in a telephone interview. The follow-up took place in May 2017.

### Outcomes

Our primary outcome was the Oxford Elbow Score (OES) of the injured arm. The OES is a validated, reliable, and responsive 12-item, three-domain (pain, function, social-psychological (s-p)) patient-reported outcome measure, specifically designed and developed for assessing outcomes of elbow surgery [8]. Secondary outcomes were the Mayo Elbow Performance Score (MEPS), Quick Disabilities of Arm and Shoulder and Hand (qDASH) and subjective satisfaction with the function of operated elbow on numeric rating scale 0 to 10 (10 fully satisfied) [9, 10]. The active and passive elbow flexion and extension were measured with a goniometer, and forearm pronation-supination was measured with a “Myrin” pro-supinometer (Medema, Solna, Sweden) held in a fist between the 2^nd^ and 3^rd^ fingers. Measurements of the range of motion and MEPS items were also obtained of the contralateral elbow of patients who attended the follow-up visit to give an internal control. Patients interviewed by telephone answered the OES questions, injury side and arm dominance. Radiographs were assessed regarding the primary treatment episode (quality of reduction, appropriate placement of implants as assessed by the senior authors, loss of reduction and complications during the primary follow-up) and at possible subsequent visits (development of osteoarthrosis, migration of implants, other complications).

### Surgeries, rehabilitation and clinical follow-up

The surgeries were performed either by or under the direct supervision of an experienced orthopaedic trauma surgeon within a few days of the injury, while open fractures were operated emergently. The usual operative protocol was general anaesthesia, lateral decubitus position with arm support and hanging forearm, ORIF with medial and lateral column fixation with anatomic locking plates placed orthogonally according to the AO principles. 3.5 mm LCP Distal Humerus Plates or 3.5/2.7 mm VA LCP Distal Humerus Plates (DePuy Synthes, Raynham, USA) were used. Computed tomography was routinely used to help preoperative planning. Olecranon osteotomies were closed with a K-wire tension band or plate. Cannulated screws, headless compression screws and bioabsorbable pins were used to fix articular fragments as necessary. Postoperative rehabilitation was guided by a physiotherapist. Passive range of motion exercises were begun immediately postoperatively, and active exercises after 3 weeks and load bearing was gradually allowed after 6 weeks. Arm sling was worn for comfort for up to 3 weeks.

During the primary treatment episodes patients were typically followed in the outpatient clinic, including radiographs, at 6 and 12 weeks. Follow-up was continued until union of the fracture was established and adequate function of the upper limb was regained.

### Statistical methods

For comparisons of means of continuous data, we used Student’s T-test to test statistical significance. Significance level was set at p ≤ 0.05. There was no missing data in the follow-up measurements.

## Results

We included 39 patients of which 14 attended full follow-up and 9 answered the telephone interview (Fig. 1). Mean follow-up time of participating patients was 3.2 years (SD 1.6; range 1 to 6.2)). Patient demographics, fracture types and injury mechanisms are presented in Table 1.

**Table 1:** Patient and treatment characteristics by follow-up status

	**All**	**OES data**	**Follow-up visit**	**Not available**
**Number (%)**	39 (100%)	23 (59%^b^)	14 (36%^b^)	16 (41%^b^)
**Age at injury**^a^	75.9 (65.3–90.2)	75.0 (65.3–86.4)	74.2 (65.3–82.8)	77.2 (65.5–90.2)
**Female**	30 (77%)	20 (87%)	13 (93%)	10 (63%)
**Fracture type**				
C1	22 (56%)	15 (65%)	8 (57%)	7 (44%)
C2	4 (10%)	4 (17%)	2 (14%)	0
C3	13 (33%)	4 (17%)	4 (29%)	9 (56%)
**Open fracture**	10 (26%)	3 (13%)	1 (7%)	7 (44%)
**Mechanisms of injury**				
Simple fall	31 (79%)	20 (87%)	12 (86%)	11 (69%)
Fall (< 3 m)	3 (8%)	1 (4%)	0	2 (13%)
Fall while cycling	4 (10%)	2 (9%)	2 (14%)	2 (13%)
Other	1 (3%)	0	0	1 (6%)

^a^mean (range). Years

^b^of total N

### Outcomes

For the 23 patients with OES data, mean OES pain was 83 (SD 17), OES function 83 (17) and OES s-p 79 (20). Mean total OES was 81 (15).

Secondary outcomes for the patients (*n* = 14) who attended full follow-up are presented in Table 2. The MEPS and the flexion–extension range of motion were statistically significantly lower in the injured elbows than uninjured sides. One patient had flexion–extension arc less than 105 degrees.

**Table 2:** Outcomes

**Primary outcome, *N* = 23**		**Injured arm**		
OES pain	score	83 (17; 44–100)		
OES function	"	83 (17; 44–100)		
OES social-psychological	"	79 (20; 31–100)		
OES Total	"	81 (15; 46–100)		
**Secondary outcomes, *N* = 14**		**Injured arm**	**Uninjured arm**	***p***‡
Active arc of motion	degrees	119 (19; 75–145)	146 (8; 135–160)	< 0.0001
Extension deficit (active)	"	22 (14; 5–60)	1 (6; -5–15)	< 0.0001
Maximum flexion (active)	"	141 (6; 130–150)	148 (5; 140–155)	0.003
Active forearm pro-supination arc	"	177 (14; 150–200)	175 (17; 140–200)	0.7
Forearm pronation (active)	"	91 (19; 70–100)	90 (12; 60–110)	0.9
Forearm supination (active)	"	86 (12; 60–100)	85 (16; 50–100)	0.9
MEPS	points	85 (17; 50–100)	100 (1; 95–100)	0.003
MEPS categories: excellent ≥ 90 / good 87–89 / fair 60–74 / poor < 60	number per category	6 / 4 / 3 / 1	14 / 0 / 0 / 0	
qDASH	points	19 (16; 2–43)		
Subjective satisfaction	NRS 0–10	9 (1; 7–10)		

Data given as mean (SD; range) or numbers

^‡^*p* comparing injured to the uninjured side

**Fig. 2:**
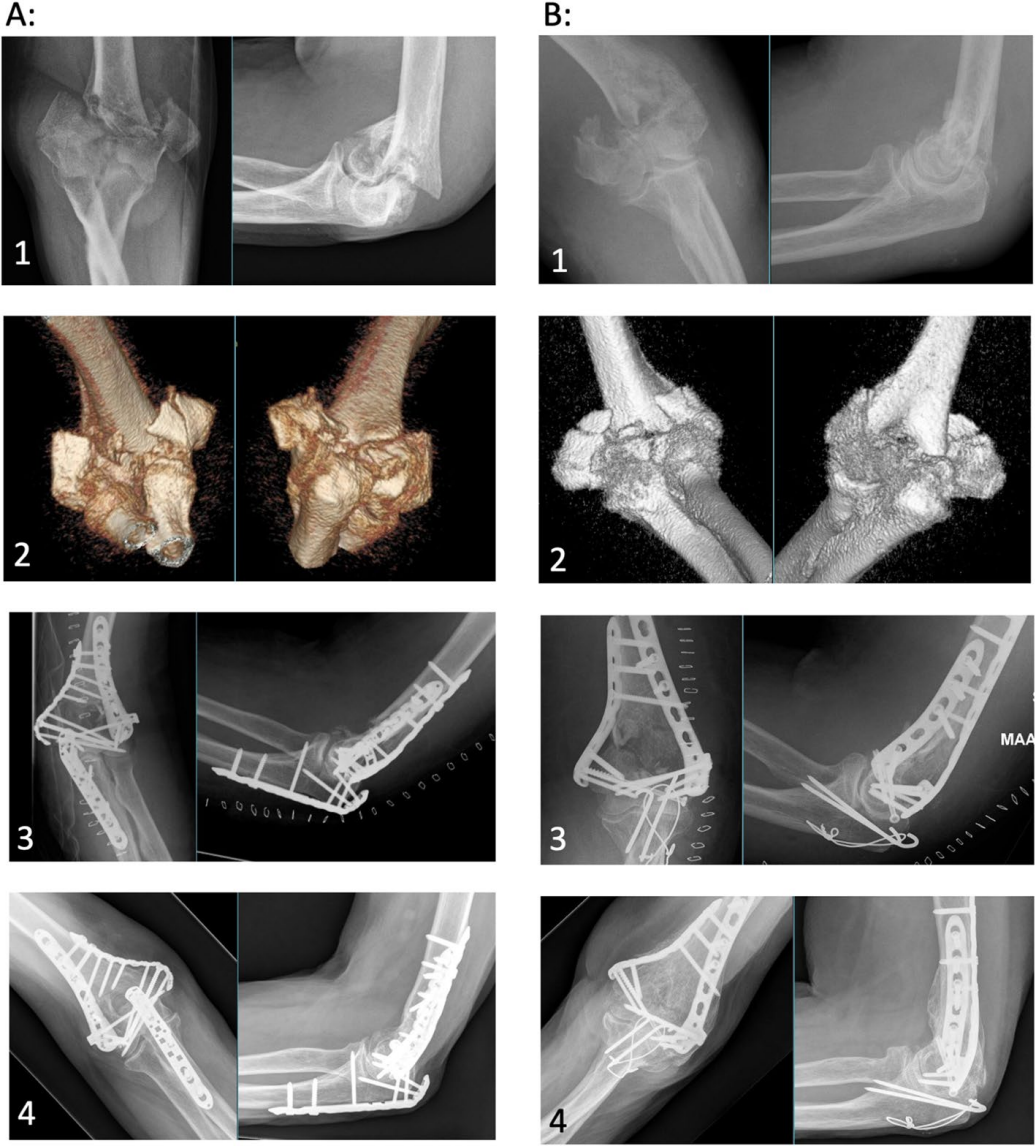
Radiographs of patients with typical fractures resulting in. **A** a good result (OES 94, flexion–extension arc 125 degrees, MEPS 100, qDASH 10) and **B** stiff elbow with moderate outcome (OES63, flexion–extension arc 75 degeres, MEPS 60, aDASH 38). 1: postinjury radiograph. 2: postinjury CT 3D reconstruction. 3: postoperative radiograph. 4: radiograph at follow-up (**A**: 1,8 years, **B**: 2,5 years)

Radiographs were obtained from all of the 14 patients who attended the follow-up visit. Four patients had developed minor osteoarthritic changes since their previous radiograph taken during the primary treatment episode. No other late complications were evident on the radiographs. Figure 2 shows the radiographs of a patient with a good result (A) and the patient with the worst range of motion (B).

Factors qualitatively associated with inferior outcomes were nerve injuries and permanent extension deficit over 40 degrees. Qualitatively, the loss of points in MEPS were almost always due to pain, not other factors. Also qualitatively, chronic pain in the elbows without nerve injuries was not associated with identifiable radiographic or surgical factors. Due to sample size and heterogeneity of data, no meaningful statistical analysis of predictors of inferior results was possible.

### Primary treatment episodes

The mean time from injury to first surgery was 2.2 (SD 2.0) days. All patients were treated with primary ORIF and bicolumnar plating. The posterior paratripicital approach with olecranon osteotomy was used in all cases except 1. 35 olecranon osteotomies were closed with K-wires and a tension band and 4 with a plate and screws. 21 surgeons were involved in the surgeries as seniors.

### Mortality, revision surgeries and complications

The 30-day mortality was 2/39 (5%) and one-year mortality 4/39 (10%). The 30-day mortality was due to causes we interpreted to be related to the injury and treatment: 1 patient died of perioperative myocardial infarction and 1 of pneumonia during post-injury stay in a rehabilitation hospital. During the follow-up period, 8 (21%) patients had died from unrelated causes, their mean lifetime after the injury was 2.9 years (SD 1.6) and their mean time from injury to patient file review was 4.3 (2.2) years.

The number of surgeries, reasons for revision surgeries and description of treatment are given in Table 3.

**Table 3:** Numbers of surgeries and reasons for revision surgeries. Outcomes are shown for patients who required non-implant removal revision surgeries related to the fracture treatment

Number of surgeries	Number of patients	Reason for revision surgeries and description of treatment
1	28	Primary operation only, no complications
2	5	Implant irritation: Late removal of tension band
	1	Implant irritation: Late removal of tension band and plates
	1	Non-union of olecranon osteotomy: Reosteosynthesis (good result: OES pain 94, function 100, S-P 100, Total 98)
	1	ORIF failure by 6 weeks: TEA (with modest result: OES pain 56, function 75, S-P 50, Total 60)
3	1	Technical difficulties in first surgery: A revision of failed ORIF, TEA at nine months from the injury (with a good result: OES pain 100, function 100, S-P 75, Total 92)
	1	Postoperative wound dehiscence: Wound revision and removal of olecranon plate five months from injury (wound healed, no outcome data, patient died during the follow-up period)
10	1	Deep infection with osteomyelitis: Removal of implants, multiple revisions, resection of osteomyelitic bone and eventually the joint. Led to an almost painless, but poorly functioning elbow (OES pain 81, function 44, S-P 31, Total 52)

There was 1 traumatic median and 2 traumatic radial nerve injuries, and 1 major iatrogenic ulnar nerve injury and 1 ulnar nerve entrapment after the surgery.

There were no minor infectious complications, nor heterotopic ossification.

10/39 (26%) patients had an open fracture, of whom 1 attended the follow-up visit and 2 answered the telephone questionnaire; 5 had died, 1 of a cause related to the injury, 1 could not be reached and 1 was not able to participate.

### Concomitant injuries and joint disease

14/39 (36%) patients had concomitant fractures. 5 patients had ipsilateral upper extremity or shoulder fractures (2 proximal humerus and 1 distal radius, 1 clavicle, 1 proximal radius), 2 patients had contralateral upper extremity fractures (proximal humerus and clavicle) 1 patient had a rib fracture and 6 had lower body fractures (2 proximal and 1 distal femur fractures, 2 had a fracture of the acetabulum and 1 proximal tibia fracture). None had pre-existing inflammatory arthritis and 2 had minor osteoarthritic radiographic changes in the fractured elbow.

Four of the 14 patients with concomitant injuries had died during the follow-up period, one within 30 days of injury. They died on average 1.4 (2.2) years post-injury. Compared to 2.9 (1.5) year average lifetime for patients without concomitant injuries who died, no statistically significant (*p* = 0.22) difference was found.

### Radiographs during the primary treatment episodes

Review of postoperative radiographs showed that 30/39 (77%) fractures had been reduced anatomically, 8 (21%) with 1-3 mm and 1 (3%) with 4-5 mm malreduction. 37 (95%) patients had appropriate placement of hardware, 2 (5%) had suboptimal plate positioning leading to short and non-interdigitating screws.

Radiographs taken during follow-up were available for 36 (92%) patients, with mean time of 6.4 months (SD 5.1; range 0.3 to 21.5) from postoperative radiograph to the last images during normal clinical follow up. Review of these showed that 21/39 (54%) fractures and osteotomies had united without complications. 8 (21%) fractures had united with no loss of reduction, but the olecranon osteotomy had widened (< 5 mm) before union. 1 (3%) patient had a non-union of the olecranon osteotomy (leading to a re-osteosynthesis of the osteotomy). All these radiographic osteotomy complications were found osteotomies closed with K-wires and a tension band, but the osteotomy widening before union was not qualitatively associated with inferior results. 6 (15%) patients had a minor secondary collapse of the distal humerus, 1 (3%) a joint-destroying avascular necrosis (leading to TEA), 1 (3%) a loss of reduction (treated with TEA) and 1 (3%) a deep infection leading to loss of reduction (which led to eventual resection of distal humerus and proximal ulna). 3 (8%) patients had minor and 2 (5%) extensive osteoarthritic changes at this point.

## Discussion

In our series, ORIF of displaced, intra-articular AO/OTA C-type distal humerus fractures of patients at least 65 years old in a high-volume trauma centre resulted in mean DASH score 19, mean MEPS 85, and mean arc of motion of 119 degrees. DASH scores were similar to those in the general population of the same age [11]. There was a statistically significant and also likely patient important difference in MEPS and arc of motion compared to the uninjured elbow. The one-year mortality was 4/39 (10%).

Strengths of our study include reliable identification of patients from the database of a large volume tertiary referral centre, the use of a validated outcome measure of function and reliable hospital record data regarding treatments and mortality. To our knowledge, this is the first published series of older adult patients treated exclusively with anatomic pre-contoured locking plate constructs. Limitations include a relatively low number of patients, lack of preoperative data of function – which we sought to mitigate by the use of the contralateral side as a control when appropriate – and retrospective design. The considerable loss to follow-up can be viewed as a weakness, but it is an inherent phenomenon in this patient group. 12 out of the 16 patients lost to follow-up had either died or were too neurologically impaired to participate. This is especially true for the open fractures where 6 out of 7 lost to follow-up had died or were too ill to participate, with only one of the deaths related to the injury (perioperative myocardial infarction). We think this to be a reflection of the “frailty fracture” nature of these injuries in the elderly, and the loss to follow-up is unavoidable in this patient group. Similar, follow-up time dependent losses to follow-up due to high mortality, up to 60%, have been reported in studies of the same patient population [1, 12–14]. The number of patients per year was relatively low, 6, and the number of surgeons high, 21. This reflects the rarity of these injuries and the daily practice in our high-volume university trauma centre, and in our view makes the results generalizable to other similar hospitals with broad expertise.

Our results are similar to recently published series and a systematic review of earlier studies regarding outcomes and complications, though the flexion arc is better in our series (Table 4) [1, 3, 15]. Also in studies which have included younger patients, the DASH scores have been similar to the respective population normal values, which is the case in our series as well [11, 16–19]. In the studies using locking plates (anatomic and reconstruction plates by Shannon et al. and only anatomic plates in our study), the results seem to be better than in studies predating the widespread use of locking plates, supporting the clinical experience that locking plate technology offers a true advantage in the treatment of these fractures. The MEPS results (85—91) and flexion arc (90 – 112) have been quite uniform regardless of the age of the patients.

**Table 4:** Outcomes in studies of similar patients

Article name (year)	Number of patients	Number at follow-up	Patient age years	F-U years	Fracture AO types	Only locking plates?	MEPS	DASH	Flex-ext arc	Sup-pron arc	Country
**Githens et al. (2013) Meta-analysis, ORIF**	-^b^	292	75^c^	3.6	B and C	No	88	35^f^	100	-^b^	Multiple
**Virani et al. (2017)** [15]	63	41	66^c^	3.2 (2.2–6.8)	C	No	85	21^ g^	105	156	India
**Shannon F et al. (2018)** [18]	21	16	78 (70–84)^d^	4 (1–8)	C	Yes	91	19	97	147	United States
**Our study**	39	23 (14 + 9)	79 (69–93)^d^	3.2 (1–6.2)	C	Yes	85	19	119	177	Finland
**TEA studies**											
**Streelzow (2021)**	40	21	79 (SD9)^e^	5 (2–13)	-^b^		90	31	111	152	Canada
**Barco (2017)**^a^ [13]	29	20	75 (38–93, SD12)^c^	> 10	-^b^		91	-^b^	94	152	United States
**Githens et al. (2013)** [3] **Meta-analysis, TEA**	-^b^	271	74	3.8	B and C		90	39^f^	101	-^b^	Multiple

Data presented as numbers, means, means (range) and means (SD)

*F-U* Ffollow-up, *Flex-Ext* Flexion–extension (arc was calculated by subtracting the mean extension loss from mean flexion if direct data was not available), *Sup-pron* Supination-pronation (arc was calculated by adding pronation and supination if direct data was not available)

^a^data from non-rheumatoid patients

^b^data not available

^c^unspecified

^d^at follow-up

^e^at index surgery

^f^data from 3 studies of 10 included in the review for ORIF, 5 studies of 13 for TEA

^g^DASH is reported arm-specifically, the value of the injured arms was tabulated

Our < 30 days and one-year mortality rates, about 5 and 10% respectively, were similar to the 2,2 and 9,1% which have been reported from a large database study from New York, USA [20]. Another large database study [21] reported low < 30 days mortality rates, 1/216, which, considering the low event rates, is similar.

In our series, 11/39 (28%) patients had at least one revision surgery. Five were minor implant removal procedures related to tension band irritation, which is lower, but similar to rates of implant removal after olecranon fracture surgery with tension band or plate fixation [22, 23]. Our rate of revision surgeries is higher than in comparative series. If tension band removals are excluded, the rate of revision surgeries is similar to other series [3]. The large number of implant removals is a reflection of our routine use of olecranon osteotomy to aid reduction of the articular fragments and low threshold for symptomatic tension band removals.

The complication rates are very similar to what others have reported, though comparisons are difficult due to varying reporting and classification of complications [3]. In our series, true non-unions of the humerus or olecranon osteotomy were rare (1 patient, 3% each). Minor collapse of the humerus (6/39, 15%) and widening of olecranon osteotomy (8/39, 21%) were relatively common, but these did not seem to affect the results. There was 1 deep infection (3%) and 1 superficial wound healing problem (3%), similar to what others have reported, 2 and 4.3% respectively. Two patients (5%) had an iatrogenic nerve injury, again similar to reported rates (6.4%) [3]. The uniform rate of complications across studies can be seen to reflect the frailty of the patients and relative to that serious injuries.

Whether locking plates offer a true advantage over non-locking constructs remains unclear. Our clinical experience, which is unfortunately hard to explore robustly, is that locking plates allow a stable osteosynthesis in more comminuted fractures than non-locking plates. This is supported by laboratory findings, in a fracture model orthogonal plating with anatomic locking plates offers a more stable osteosynthesis in bone with low mineral density [5]. There also was a consistent difference of about 20 degrees more flexion in our series to previous studies. Whether this is due to the new plate technology allowing more aggressive rehabilitation or some other factor cannot be reliably answered. There were no complications specific to locking plates, and in contrast to some series, the rate of fixation failure was low in our series [18].

As an implication to practice, we think our results support the strategy of treating fractures amenable to fixation primarily with ORIF and reserving TEA for the very comminuted fractures and as a second-line option should the primary ORIF fail. The results appear to be similar (Table 4), but the complication profile of ORIF is more benign than in TEA [3]. During the study period, we used primary TEA for five patients (all with C3 type fractures). However, it has to be noted that our results are from a high-volume trauma centre with upper extremity surgeons experienced in both acute trauma care and elective arthroplasty – in different settings the available expertise has to be considered in addition to the characteristics of each patient. In practice, also the probable limited lifetime of up to half of the patients has to be considered, and the treatment should be as straightforward and low complication risk as possible.

Topics for future research include how to avoid revision surgeries in the form of implant removals. A randomized trial comparing different fixation methods of olecranon osteotomies, perhaps including all-suture osteosynthesis among the treatment options would be clinically relevant, and considering the large proportion of patients who undergo implant removal, also likely feasible [24]. We think that an RCT comparing ORIF to TEA is unlikely to be fruitful due to the rarity of these injuries and the similar results between the groups in the one published RCT and available non-comparative series [25]. The required large sample size to show superiority is likely to be unfeasibly large. In the light of our and previous results, obtaining superior results with a TEA to locking plate ORIF in C1 and C2 fractures seems very unlikely and C3 fractures are rare. The complication profiles and how soon the function reaches adequate levels, and possible predicting factors, might be assessed through registry studies or large, multi-centre retrospective studies.

## Conclusions

Our results indicate that “reducible” distal humerus fractures in older adults can be treated reliably with ORIF in a high-volume trauma centre using anatomic locking plates. The expected result is return of function allowing activities of daily living, with some residual pain and a moderate extension deficit and moderate loss of function. About one in four patients can be expected to undergo at least one additional surgery.

## Data Availability

The original pseudonymised data workbooks are available from the corresponding author on a reasonable request. Please send email to tuomas.lahdeoja@hus.fi.

## Abbreviations

OES: Oxford Elbow Score
MEPS: Mayo Elbow Performance Score
ORIF: Open reduction and internal fixation
TEA: Total elbow arthroplasty
